# Domestic

**Published:** 1841-07-17

**Authors:** 


### DOMESTIC.

*Navy Department, July* 9, 1841.

The Board of Naval Surgeons recently convened in the city of Philadelphia have closed their proceedings, and reported the result to the Department. Of the Assistant Surgeons examined, the following have been found qualified for promotion, viz.:—Charles A. Hassler, of the date of 1834; David Harlan, of the date of 1835; Victor L. Godon, do. do.; Robert Woodworth, do. do.; J. Dickinson Miller, of the date of 1836.

Of those examined for admission into the Navy as Assistant Surgeons, the following have been found qualified, viz.:—Andrew H. Henderson, Ellis Hughes, John Hastings, Charles H. Broughton, R. T. Maxwell, Edward McKinley, Alexander Y. P. Garnett.

---

*Massachusetts General Hospital—Surgical Cases treated by* S. D. Townsend, M. D., Surgeon.

*Warty Excrescences of the Lip.*—An Irishman, aged 61 years, entered the house May 28. About a year and a half since, first perceived a small white speck on the edge of lower lip, near the right angle, which he disregarded till four months since, when it had increased to the size of a small pea. There was no pain attending it, but the cuticle frequently pealed off, leaving a raw surface. At present the right angle of lower lip is more than twice the natural size, with ragged and unhealthy ulcerations. Tumor occupies about a quarter of the lip. He has latterly visited the Hospital as an out-patient frequently, and has had the carb. and phosphat. of iron, and equal parts of acetate of copper and powdered savin leaf applied to the excrescence, with some benefit.

The operation for the removal of the disease was performed in the following manner: A straight bistoury was passed through the cheek from within outwards, at a point three quarters of an inch from right angle of the mouth and a little below, and brought out at the angle of the mouth; another incision was then commenced at the bottom of the first, brought around and including the tumor upward to the edge of the lip, about an inch from angle of mouth. This irregularity in the incision and deviation from the usual form of the letter V, was rendered necessary by a part of the disease being situated external to the angle of the mouth. There was but little bleeding from the wound, which was brought together by three stitches and compresses, wet in cold water, kept constantly applied to it. The mouth was considerably diminished in circumference and misshapen by this operation, owing to the peculiar situation of the disease. Union seemed to be effected until the fourth day, when the stitches were removed, and a separation took place for about one third of an inch. Some hardness about the wound and appearance of fungus at the edge of the ununited part presented itself, which disappeared on the repeated application of the nitrate of silver. On the 18th day he was discharged well, with a mouth well shaped.

The second case is a seaman, aged 70 years. About four years ago, he first perceived a small, black, indurated spot on the edge of the under lip, not tender to the touch. The cuticle frequently pealing off, left a raw, bleeding surface. It was not painful. Latterly it began to increase gradually, and gave him considerable inconvenience. At the period of his admission into the Hospital, the disease covered the upper part of the lower lip, and a hard, irregular tumor extended nearly to the corners of the mouth on each side. A fortnight previous, a fungous growth appeared in the centre, which was treated with alum, since which an ulcer three quarters of an inch long has appeared on the edge of the lip, painful on exposure to air, and discharging a thin, serous fluid.

The operation was performed by Dr. Hayward* in the following manner: An incision in the form of the letter V was made by two strokes of the scalpel, obliquely from the edge of the lip to the chin, removing a triangular piece upon which the tumor was situated, of about half the length of the lip. The edges of the wound were then brought together by three stitches, and compresses wet with cold water directed to be applied constantly to the wound. On the fifth day from the operation the stitches were removed, and the wound was perfectly united.

An inquiry has generally been made of patients with this disase of the lip, as well as in cancerous affections of the tongue, whether they were in the habit of using tobacco. In both these cases they were accustomed to smoke a short pipe, and in most of those which I have seen the disease has occurred in elderly people who gave a preference to the pipe over the cigar; it suggested itself to my mind whether the heat from the earthen material may not have greatly aided in producing the disease. The term cancer seems to be incorrectly used when applied indiscriminately to this affection, as it differs from that disease in many points, not always affecting the absorbent glads at a distance, and, besides, it admits of a cure in many instances, and under different modes of treatment.—*Bost. Med. and Surg. Jour.*

* By a mutual agreement, such a distribution is made, that the surgeon in attendance gives an opportunity to each of his colleagnes to operate occasionally throughout the year.

